# Rumen microbiota and fermentation parameters in Tibetan semi-fine wool sheep reflect growth stages and potential nutritional adaptations

**DOI:** 10.5713/ab.250616

**Published:** 2026-02-06

**Authors:** Hongjin Liu, Jiahui Hao, Xueping Han, Shixiao Xu, Xungang Wang, Qian Zhang, Tongqing Guo, Chongliang Luo, Linyong Hu, Wenmin Zhang

**Affiliations:** 1 Northwest Institute of Plateau Biology, Chinese Academy of Sciences, Xining, China; 2 University of Chinese Academy of Science, Beijing, China; 3 Qinghai Agri-animal Husbandry Vocational College, Xining, China; 4 Langfang Normal University, Langfang, China

**Keywords:** 16S rRNA, Adaptation, Age, Rumen Microbiota, Semi-fine Sheep, Short-chain Volatile Fatty Acids

## Abstract

**Objective:**

The rumen microbiota plays a pivotal role in fermenting plant biomass, which is essential for nutrient conversion. Although extensive research has examined the intestinal microbiome of Tibetan livestock, the rumen microbiota of semi-fine wool sheep (SF-sheep) remains poorly characterized. This study aimed to profile age-related changes in the rumen microbiota and fermentation parameters of SF-sheep to uncover potential microbial-mediated adaptations to different growth stages.

**Methods:**

Full-grazing male SF-sheep were randomly assigned into three age groups: two-month-old lambs, yearling sub-adults, and approximately 50-month-old adults. 16S rRNA gene sequencing and high-performance liquid chromatography were used to analyze the rumen microbiota composition and short-chain volatile fatty acids (SCVFAs).

**Results:**

Age-dependent differences were observed in ruminal NH_3_-N concentrations, with sub-adult sheep exhibiting higher levels than young and adult individuals, whereas SCVFAs concentrations remained relatively stable. With age, the rumen microbial community structure tended to become more homogeneous, whereas microbial diversity and complexity showed a marked increase during adulthood. At the phylum level, Saccharibacteria and *Succiniclasticum* were enriched in sub-adults, whereas Euryarchaeota and *Prevotellace_UCG_001* were more abundant in adults; no microbial biomarkers were detected in young sheep. Correlation analyses indicated that age and NH_3_-N concentrations were the primary factors shaping the rumen microbiota. SCVFAs, including acetate, butyrate, and propionate, were positively associated with fibrolytic and polysaccharide-degrading bacteria such as *Prevotella_1, Treponema_2*, and *Selenomonas_1*. The rumen microbial communities were classified into two enterotypes. Enterotype 1, predominantly observed in young SF-sheep, showed higher abundances of Kyoto Encyclopedia of Genes and Genomes Orthologs (e.g., K00656, K00239, K01966) associated with acetate, propionate, and butyrate synthesis.

**Conclusion:**

The rumen microbial ecosystem of SF-sheep showed pronounced age-dependent restructuring in microbial composition and diversity, accompanied by nitrogen metabolism. These changes represent dynamic microbial adaptation to the host’s physiological state and potential developmental shifts in nutrient requirements, offering valuable insights for age-specific nutritional management strategies.

## INTRODUCTION

The rumen is the most crucial organ of ruminants, playing a key role in nutritional metabolism, including fiber degradation, energy provision, and protein balance through microbial fermentation [1]. Recent studies have highlighted that the interactions between rumen microorganisms and their hosts are essential for host performance and physiological function [2]. A prime example of these relationships occurs in the first chamber of the ruminant gastrointestinal tract—the rumen. Here, plant biomass is exposed to a variety of microorganisms that ferment plant fibers into volatile fatty acids, providing stable and favorable energy and nutrition for microbial growth. Bacteria form the largest part of the microbial composition in the rumen and are susceptible to environmental variations, such as host genetics, diet, altitude, etc. [3]. Extensive studies on various hosts, including sheep, cattle, goat, buffalo, Tibetan sheep, and yak have provided reliable data to support rumen microbial-mediated processes in forage regulation, nutrient absorption and body immune function in ruminants [4,5].

Qinghai semi-fine-wool sheep (SF-sheep) is a breed locally developed on the Qinghai-Tibet Plateau (QTP). Valued for its rapid growth, high-quality wool, and meat production, it constitutes a major economic resource for local pastoralists. Studies have shown that their remarkable traits were found to be associated not only with the maternal genetic influence but also with the composition of intestinal microorganisms [6]. The SF-sheep live around the Chaka Salt Lake in the northeastern part of the QTP (at an altitude of over 3,400 m), and have long adapted to extreme environments such as low oxygen partial pressure, strong ultraviolet radiation and large temperature differences between day and night in high-altitude areas. Their main food is the salt-tolerant and alkali-tolerant plants around the Salt Lake (such as *Suaena glaurata*, *Citronella cinerata*, and *Cymbidium goeringii*, etc.), which are rich in minerals (such as sodium salts, magnesium, and calcium) and special secondary metabolites (such as polyphenols and alkaloids), and are significantly different from the feed of other ruminants in QTP.

The age of an animal is a crucial physiological trait that strongly influences the composition of rumen microbial community [7,8]. Studies have shown that aerobic and facultative anaerobic microorganisms rapidly colonize the rumen immediately after birth [9]. As animals mature, these microorganisms are progressively replaced by anaerobic species acquired from parents and the environment, with the microbial community reaching a stable state by 6 to 8 weeks of age [10]. In the sub-adult and adult stages, rumen microbes ferment dietary components, particularly indigestible plant cellulose, to generate short-chain volatile fatty acids (SCVFAs), microbial protein, ammonia, and other metabolites [10]. Therefore, understanding the influence of age on the rumen microbiota is essential for optimizing ruminant production systems, improving animal health, and developing strategies to enhance nutrient utilization and feed efficiency. However, research on the rumen microbiota of ruminants on the QTP has primarily examined factors such as feeding regimes, sex, and altitude. Studies specifically addressing the effects of age on the rumen microbiota of Qinghai SF-sheep remain limited. Existing research on this breed has primarily focused on the bacterial composition along the intestinal tract, leaving the developmental dynamics of the rumen microbial ecosystem largely unexplored [6].

Despite the importance of the rumen microbiome for nutrient utilization, key gaps persist in our understanding of the SF-sheep. Specifically, it remains unknown how its rumen microbial community assembles and functions across critical developmental stages (young, sub-adult, adult) under the unique pressures of year-round grazing on saline-alkaline pastures at high altitude. To address these gaps, in the present study, gas chromatography and high-throughput sequencing technologies were employed to analysis SCVFAs and rumen microbiota structure in lambs, sub-adults, and adult SF-sheep. This research aims to characterize the rumen microbiota and SCVFAs across different age groups of SF-Sheep. Furthermore, we investigate age-related similarities and differences in microbial community composition and function. Our findings provide valuable information for nutritional regulation and the production strategies for Qinghai SF-sheep on the QTP.

## MATERIALS AND METHODS

### Experimental design and ethics approval

In this study, only male sheep were selected as experimental subjects to minimize the influence of sex-related physiological differences on the rumen microbiota and fermentation characteristics. Female sheep may display distinct hormonal profiles and growth patterns that could introduce additional variability, potentially confounding the interpretation of age-related changes. By restricting the study to male sheep, we aimed to ensure greater consistency and reliability of the data. A total of 47 male SF-sheep were randomly selected in June 2018. All the sheep grazed on the same pasture under a free-range system, with no supplementary feeding, although water was available *ad libitum*. The sheep were divided into three age groups: 15 two-month-old lambs (abbreviation LS; average weight: 11.50±2.30 kg), 16 yearling sub-adults (SS; average weight 44.21±8.32 kg), and 16 approximately 50-month-old adults (AS; average weight 77.54±9.65 kg).

### Forage and rumen fluid collection

The method of herbage collection followed a previously described protocol [11]. Briefly, eight quadrats measuring 50×50 cm were randomly placed in areas of grass showing clear signs of grazing, with approximately 10 meters between each quadrat. The aboveground biomass was clipped using scissors, and non-edible plants were removed prior to nutrient analysis to ensure an accurate assessment of forage quality.

Rumen fluid was conducted following procedures described in previous studies [2]. Rumen fluid was collected between 08:00 and 10:00 to standardize sampling relative to grazing activity and minimize diurnal variation in fermentation parameters. Approximately 25 mL of rumen fluid was obtained using an esophageal tube equipped with a specialized exhaust tube inserted through the mouth into the rumen. The fluid was then filtered through four layers of sterilized gauze, after which 2 mL was transferred into sterilized cryogenic tubes and immediately stored in liquid nitrogen for subsequent DNA extraction. In addition, approximately 20 mL of rumen fluid was stored in a portable refrigerator at 4°C for the determination of rumen fermentation parameters, including pH, ammonia nitrogen (NH_3_-N), and SCVFAs.

### Forage nutrition composition analysis

Crude protein (CP) and ether extract (EE) contents were determined following AOAC method 988.05 [12] and the diethyl ether extraction was determined by Soxhlet method as described in AOAC method 2003.5 [12], respectively. Acid detergent fiber (ADF) and neutral detergent fiber (NDF) were analyzed using the methods described by Van Soest et al [13]. The nutritional composition of the forage was as follows: CP, 12.53±0.51%; crude fat, 14.97±0.67 g/kg; NDF, 63.30±1.65%; and ADF, 36.93±1.56%.

### Rumen fermentation parameters measurement

Upon sample collection, the pH of the rumen fluid was immediately measured using a portable pH meter (PHSJ-3F; Precision Instruments Company). The concentrations of SCVFAs were determined according to the method established by Liu et al [2].

### DNA extraction, polymerase chain reaction amplification, and 16S rRNA sequencing

Microbial DNA was extracted using the E.Z.N.A. stool DNA Kit (Omega Bio-tek) following the manufacturer’s protocols. The hypervariable V3-V4 region of the 16S rRNA was amplified by polymerase chain reaction (PCR) with an initial denaturation at 95°C for 2 min, followed by 27 cycles of denaturation at 98°C for 10 s, annealing at 62°C for 30 s, and extension 68°C for 30 s. A final extension was performed at 68°C for 10 minutes. The primers used were 341F (CTACGGGNG GCWGCAG) and 806R (GGACTACHVGGGTATCTAAT). Each sample was assigned a unique eight-base barcode sequence, and PCR amplification was performed in duplicate. Detailed procedures of PCR amplification and Hiseq sequencing have been previously documented [14].

To obtain high-quality clean reads, raw sequences were filtered by removing reads containing more than 10% unknown nucleotides (N) and those with fewer than 80% of bases having a quality score (Q-value) above 20. The paired-end clean reads were then merged into raw tags using FLASH (ver. 1.2.11; http://ccb.jhu.edu/software/FLASH/) with a minimum overlap of 10bp and a maximum mismatch error rates of 2%. Noisy sequences in the raw tags were further filtered using QIIME (ver. 1.9.1; https://qiime.org/) to obtain the high-quality clean tags [15].

The clean tags were then screened against a reference database for chimera detection using the UCHIME algorithm (http://drive5.com/uchime/uchime_download.html) [16]. All identified chimeric sequences were removed, leaving effective tags for downstream analysis. The effective tags were clustered into operational taxonomic units (OTUs) at ≥97% sequence similarity using the UPARSE pipeline (https://github.com/wmaier/uparse) [17]. OTUs were taxonomically annotated using the Ribosomal Database Project classifier (ver. 2.2) based on the SILVA database (https://www.arb-silva.de/) [18]. To ensure consistent sampling depth, each sample was rarefied to 35,000 reads. Subsequently, alpha diversity indices, including Chao1, Shannon, and Sobs, were calculated using QIIME. Functional predictions of OTUs were performed using Tax4Fun (ver. 1.0) to infer bacterial metabolic pathways according to the Kyoto Encyclopedia of Genes and Genomes (KEGG) [19].

### Statistical analysis

Statistical analyses were conducted using R (ver. 4.0.2). Data normality was assessed using the Shapiro-Wilk test (*shapiro.test*). For normally distributed data, one-way analysis of variance (ANOVA) was performed using the *aov* function from the *multicomp* package (ver. 1.4.20), followed by multiple comparisons with *lsdTest* function. Non-normally distributed data were analyzed using the Kruskal-Wallis test for multiple groups, with Dunn’s post-hoc test for pairwise comparisons. Statistical significance was set at p<0.05, and adjusted p-values were reported where appropriate.

Principal coordinates analysis (PCoA) based on weighted UniFrac distances at the OTU level was performed using the *vegan*, *ade4* and *ggplot2* packages. The homogeneity of dispersions for the distance metric was then tested to identify variance differences among treatments using 999 permutations of the *betadisper* function in the *vegan*. Venn diagrams were generated with the *venn.diagram* function from the *VennDiagram* package to visualize unique and shared OTUs. Linear discriminant analysis effect size (LEfSe) was applied to identify significant microbial biomarkers using the Tutu Cloud Platform (http://cloudtutu.com.cn/). Co-occurrence networks were constructed using the random matrix theory via the Molecular Ecology Networks Analysis pipeline (http://ieg2.ou.edu/MENA), with detailed procedures described elsewhere [11].

The influence of rumen fermentation parameters on microbial community structure was examined using Mantel tests, and results were plotted with the *ade4* and *ggplot2* packages. Variance partitioning analysis (VPA) was performed using *vegan* and *ggplot2* to quantify the contribution of environmental factors (i.e., rumen fermentation parameters) to microbial community composition. Finally, canonical correspondence analysis (CCA) was conducted in Canoco 5 to explore relationships between environmental variables (age and rumen fermentation parameters) and microbial communities.

## RESULTS

### Rumen fermentation parameters characteristic among different age stages

**Figure 1:**
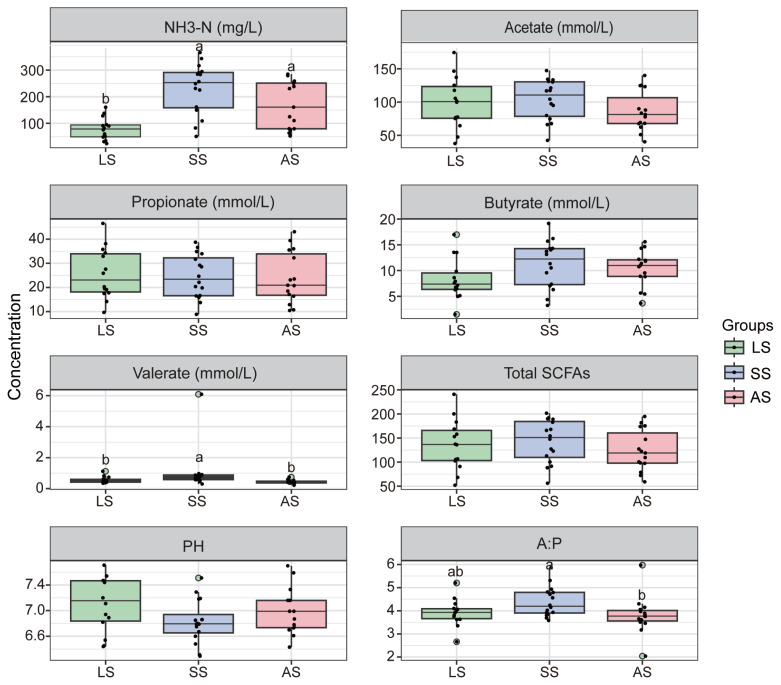
Concentrations of rumen fermentation parameters at different growth stages. The ages of 2-month-old, yearling, and 50-month-old SF-sheep are abbreviated as LS, AS, and SS, respectively. These abbreviations are used throughout the text. ^a,b^ Different lowercase letters above columns indicate statistical differences at p<0.05. Values in the same row with different letters differ significantly (p<0.05), whereas columns without letters indicate no significant differences (p>0.05). SF-sheep, semi-fine wool sheep.

The concentration of NH_3_-N varied significantly among the three age groups, with sub-adult (SS) sheep exhibiting significantly higher levels than both young (LS) and adult (S) sheep (p<0.05). The acetate-to-propionate (A:P) ratio also differed significantly, with SS sheep showing higher values than AS sheep (p<0.05). Among the SCVFAs, only valerate concentration was significantly elevated in SS sheep compared to the other groups, whereas no significant differences were observed for other SCVFAs across age groups (Figure 1).

### Overall rumen microbiota composition and core microbiota across age stages

**Figure 2:**
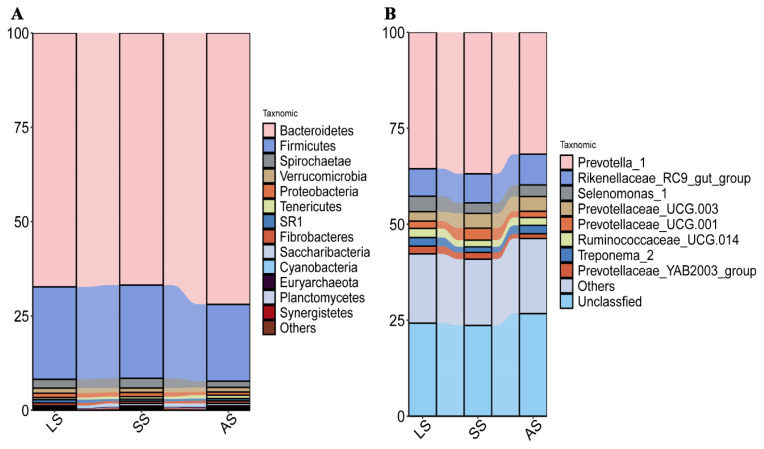
The rumen microbiome composition at phylum (A) and genus (B) level. Only taxa with a mean relative abundance of greater than 0.1% (phylum) or 1% (genus) are shown.

A total of 3,860,825 paired-end reads were obtained across all samples (mean = 82,145; standard error = 2,828). Taxonomic classification showed that the rumen microbiota was dominated by Bacteroidetes (mean relative abundance = 68.73%), Firmicutes (23.15%), Spirochaetae (2.18%), Verrucomicrobia (1.24%), and Proteobacteria (1.03%), followed by less abundant phyla (mean relative abundance<1%), including Tenericutes, SR1, and Fibrobacteres (Figure 2A). At the genus level, the five most prevalent genera were *Prevotella_1* (34.69%), *Rikenellaceae_RC9_gut_group* (7.60%), *Prevotellaceae_UCG-003* (3.41%), *Selenomonas_1* (3.23%), and *Prevotellaceae_UCG-001* (2.22%) (Figure 2B).

**Figure 3:**
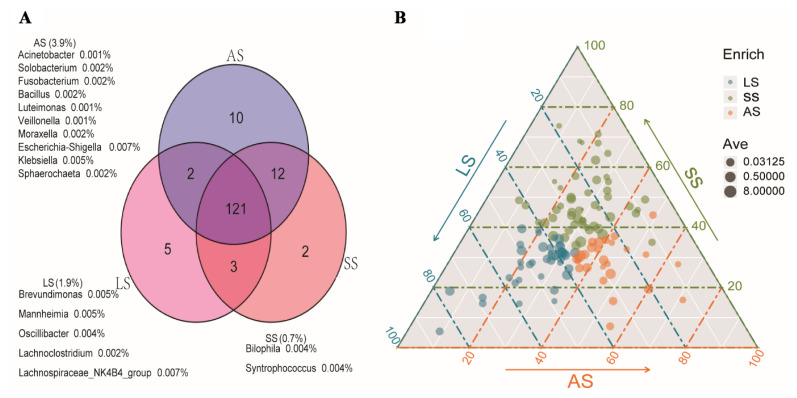
Core bacterial community and structural shifts among age groups. (A) Venn diagram at the genus level showing age-specific genera for each group, along with their average relative abundances. (B) Ternary plot of 121 rumen core genera across the three age groups. Point size indicates the relative abundance of each genus, while proximity to a vertex reflects enrichment in the corresponding age group. Circle colors denote different age groups. The ages of 2-month-old, yearling, and 50-month-old semi-fine wool sheep (SF-sheep) are abbreviated as LS, AS, and SS, respectively.

At the genus level, core bacteria—defined as taxa shared by 100% of samples—were identified based on their composition and abundance [20]. Venn diagram revealed that 121 genera were presented in all samples across the three age groups, representing 46.7% of all 259 genera (Figure 3A). Five genera were unique to the LS group—*Brevundimonas*, *Lachnospiraceae_NK4B4_group*, *Oscillibacter*, *Lachnoclostridium*, and *Mannheimia*—with average relative abundances ranging from 0.002% to 0.007%. Two genera were specific to the SS group—*Bilophila* and *Syntrophococcus*—each with an average relative abundance of 0.004%. Ten genera were specific to the AS group—*Acinetobacter*, *Solobacterium*, *Fusobacterium*, *Bacillus*, *Luteimonas*, *Veillonella*, *Moraxella*, *Escherichia-Shigella*, *Klebsiella,* and *Sphaerochaeta*—with average relative abundances ranging from 0.001% to 0.007%. A ternary plot further illustrates the distribution patterns of the dominant enriched genera. Notably, with increasing age, the proportion of core microbiota became more enriched in the SS group (Figure 3B).

### Rumen microbial diversity across age stages

**Figure 4:**
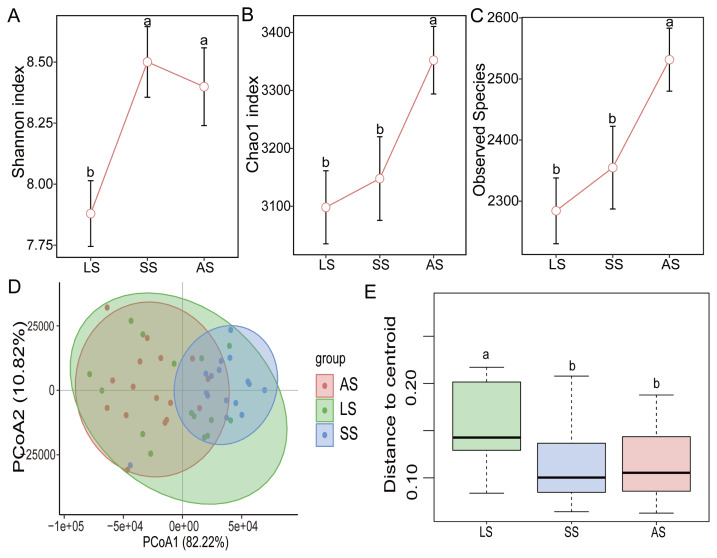
Diversity metrics of the rumen microbiota across different growth stages. Shannon diversity index (A), Chao1 richness index (B), and observed species count (C) are displayed as line charts. Principal coordinates analysis (PCoA) was conducted to evaluate overall community structure (D) and group dispersion homogeneity (E). Statistical significance was assesed using permutational analysis of multivariate dispersions (PERMDISP). The ages of 2-month-old, yearling, and 50-month-old semi-fine wool sheep (SF-sheep) are abbreviated as LS, AS, and SS, respectively. Different lowercase letters (a and b) above the line graphs and box plots indicate statistical differences at p<0.05, whereas the same letter denotes no significant difference.

The rumen microbiota in the AS group exhibited higher Shannon diversity (Figure 4A), Chao1 diversity (Figure 4B), and observed species (Figure 4C) compared with the LS and SS groups (p<0.05). For beta diversity, PCoA showed distinct age-related clustering of bacterial communities (Figure 4D), with the LS group significantly separated from both the SS and AS groups (Figure 4E).

### Differences in rumen microbial composition across age stages

**Figure 5:**
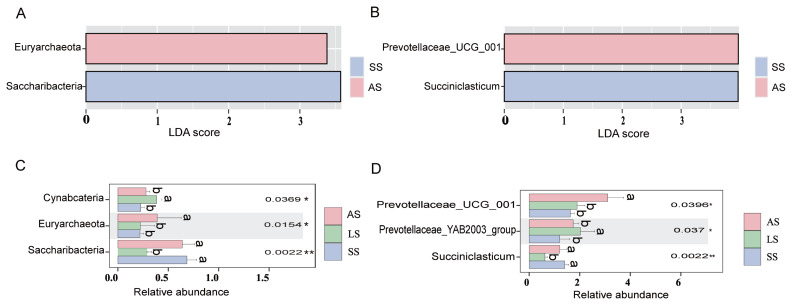
Differences in the rumen microbiota across developmental stages. Linear discriminant analysis (LDA) effect size (LEfSe) results identified significant bacterial biomarkers at the phylum (A) and genus (B) levels (LDA>2, p<0.05). Metastat analysis further validates these biomarkers at the phylum (C) and genus (D) levels. p-values are shown above the histograms. The ages of 2-month-old, yearling, and 50-month-old semi-fine wool sheep (SF-sheep) are abbreviated as LS, AS, and SS, respectively. Different lowercase letters (a and b) above the bar charts indicate statistical differences at p<0.05, whereas the same letter denotes no significant difference. Asterisks indicate significant differences for group comparisons (* p<0.05, ** p<0.01).

Using LEfSe analysis, we identified significant bacterial biomarkers at both the phylum (Figure 5A) and genus (Figure 5B) levels. Saccharibacteria and *Succiniclasticum* were more abundant in SS group, whereas Euryarchaeota and *Prevotellaceae_UCG_001* were more enriched in the AS group. No microbial biomarkers were detected in the LS group. Metastat analysis corroborated these findings and further revealed that the relative abundance of Cynobacteria (Figure 5C) and *Prevotellaceae_YAB2003_group* (Figure 5D) were significantly higher in the LS group as well.

### Differences in co-occurrence network structures across age stages

**Figure 6:**
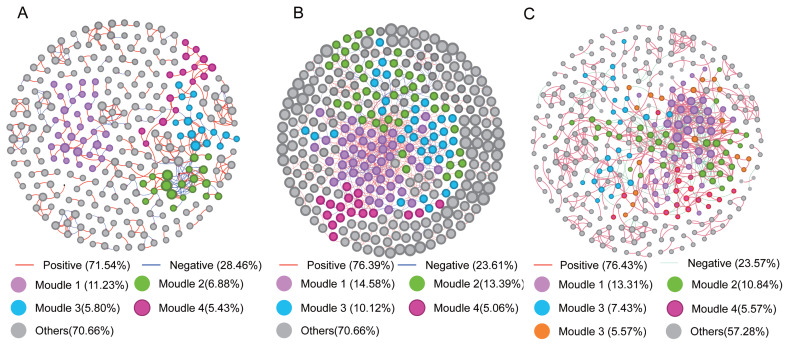
Intradomain network structure of the rumen microbiota across age stages. Topological features of the microbial co-occurrence network in two-month-old (A), sub-adult (B), and adult SF-sheep (C) were constructed based on the relative abundances of rumen bacteria at the genus level. Circle colors indicate different module hubs, and circle size reflects the relative abundance of each genus. Modules containing five or more nodes are displayed in various colors, whereas smaller modules are shown in gray. A straight line between circles represents a significant correlation (p<0.05; Spearman correlation). Positive correlations are represented by red links, and negative correlations by gray links. SF-sheep, semi-fine wool sheep.

Co-occurrence networks were constructed to identify potential microbial associations across different growth stages (Figure 6). Overall, positive correlations were the predominated among rumen microorganisms in all groups, accounting for approximately 75% of all interactions. Microbial interactions in the LS group were the simplest. In contrast, as the host aged, the microbial community became increasingly complex, as reflected by increases in the number of nodes, links, average clustering coefficient (avgCC), average degree (avgD), average path length (avgPL), and network modularity (Figure 6; Table 1).

### Relationships between rumen microbiota and rumen fermentation parameters across age stages

**Figure 7:**
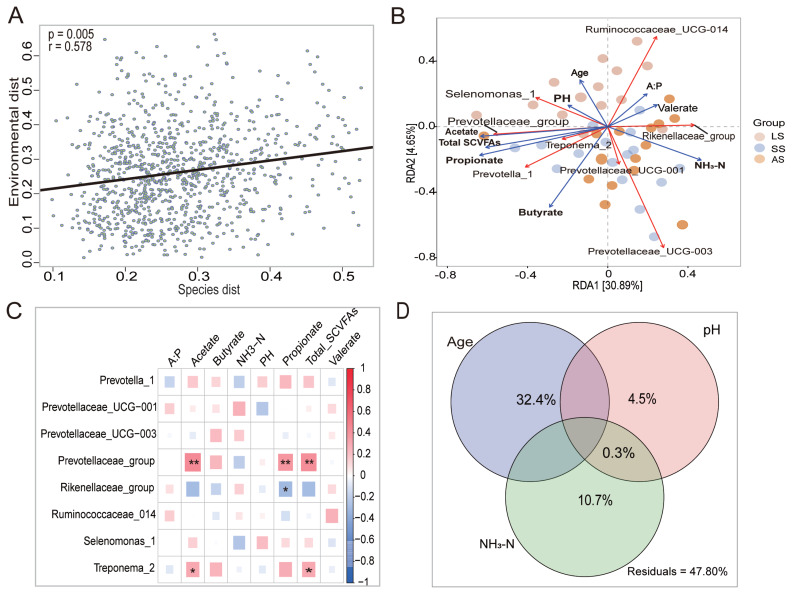
Statistical analysis of the relationship between the rumen bacterial community and rumen fermentation parameters. (A) Mantel test evaluating the correlation between the distance matrix of fermentation parameters and the distance matrix of genus abundances, based on Bray–Curtis dissimilarity. (B) Redundancy analysis (RDA) of genera in relation to rumen fermentation parameters. (C) Spearman correlation heatmap between rumen bacterial communities and fermentation parameters. (D) Variation partitioning analysis (VPA) showing the contribution of different factors to the variation in rumen bacterial community structure. The top eight genera with relative abundances greater than 1% in at least one group were included in the RDA, Spearman, and VPA analyses. The genera Rikenellaceae_RC9_gut_group and Prevotellaceae_YAB2003_group were abbreviated as *Rikenellaceae_group* and *Prevotellaceae_group*, respectively. The ages of 2-month-old, yearling, and 50-month-old semi-fine wool sheep (SF-sheep) are abbreviated as LS, AS, and SS, respectively. * p<0.05, ** p<0.01. A:P, actate-to-propionate ratio; SCVFAs, short-chain volatile fatty acids.

Mantel test analysis (Figure 7) revealed a significant correlation between rumen fermentation parameters and the rumen microbiota community structure (p = 0.005, r = 0.578). Redundancy analysis indicated shifting correlation patterns with age. Notably, NH_3_-N concentration was positively correlated with *Prevotellaceae_UCG-001*, *Prevotellaceae_UCG-003*, *Rikenellaceae_RC9_gut_group*, and *Ruminococcaceae_014*. Most of SCVFAs, such as acetate, butyrate and propionic, showed positive correlation with fibrolytic and plant polysaccharide degradation bacterium, including *Preovotella_1*, *Treponema_2*, and *Selenomonas_1* (Figures 7B, 7C). VPA indicated that 52.20% of the variation in bacterial community structure could be explained by three major variables. Age, NH_3_-N, and pH could independently explain 32.4%, 10.5%, and 4.5% of the variance, respectively, suggesting that age and rumen fermentation parameters of NH_3_-N were the dominant factor influencing the rumen microbial community (Figure 7D).

### Rumenm microbiota enterotypes and functional context with ages

**Figure 8:**
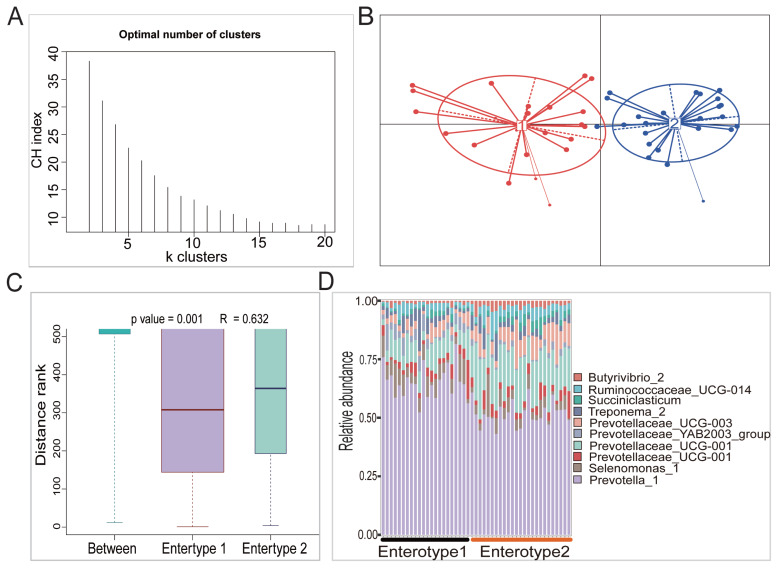
Enterotype distribution of SF-sheep rumen microbiota across different ages. (A) The optimal number of clusters for intestinal type as identified by partitioning around meidoid (PAM). (B) Variations in rumen microbiota enterotypes among different host age groups were asssed using Bray-Curits dissimilarity. (C) Differences in microbial community structures among enterotypes were evaluated using analysis of similarities (ANOSIM). (D) The relative abundance distribution of the top 10 bacterial taxa in each enterotype and their cumulative contribution rate (exceeding 70%) were analyzed using the Mann–Whitney U test with FDR-corrected p-values, as shown in Supplement 1. SF-sheep, semi-fine wool sheep; FDR, false discovery rate.

Building on previous evidence that enterotypes exhibit distinct functional characteristics [21], we investigated whether the rumen microbiota differntiates into functionally distinct clusters in response to age growth (Figure 8). Calinski–Harabasz (Figure 8A) and principal component analysis (Figure 8B) revealed that with age growth the rumen microbiota formed two distinct enterotype clusters. Notably, among the 23 enterotype 1 samples, 10 were from the LS group (Figure 8C). The clusters were distinguished by differences in the relative abundances of their representative bacterial genera. *Prevotella_1* and *Selenomonas_1* characterized enterotype 1, whereas *Prevotellaceae_UCG_003* and *Ruminococcaceae_UCG_14* characterized enterotype 2 (Figure 8D).

**Figure 9:**
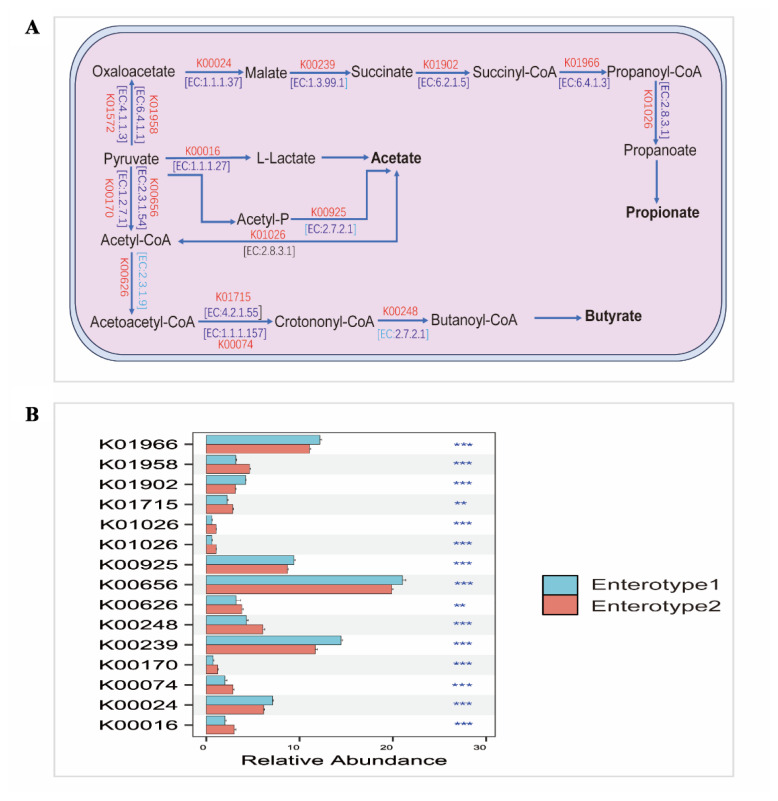
Reconstruction of the metabolic pathways based on different enterotype. (A) The metabolic pathways of SCVFAs synthesis. (B) Significant analysis of KOs that participate in acetate, propionate, and butyrate synthesis. Enzyme Commission (EC) means enzyme numbers involve the formation of each by-product. ** p<0.01. *** p<0.001. SCVFAs, short-chain volatile fatty acids; KO, Kyoto Encyclopedia of Genes and Genome Orthology.

The age distribution of of ruemn microbial enterotypes let us to hypothesis the fixed enterotype, represent by their respective microbial communities, plays a vital role in regulating nutrational requirements under the harsh saline-alkali feeding environment. To test the hypothesis, we reconstructed the metabolic pathways of the biosynthesis of SCVFAs, including acetate, propionate and byturate (Figure 9). Totally,15 KEGG Orthology (KO) numbers were observed. Notably, the top five most abundant KOs—K00656, K00239, K01966, K00925, and K00024—were enriched in enterotype1.

## DISCUSSION

### The NH_3_-N concentration in the rumen varied among different developmental stages, while short-chain volatile fatty acids concentration was relative stale

NH_3_-N is used as an indicator of the rumen environment and serves as the primary nitrogen source for the growth of rumen microorganisms [22]. It has been demonstrated that rumen NH_3_-N concentration remains high from birth to eight weeks and then continuously decreases into adulthood [23]. Our data showed that NH_3_-N concentration peaked in sub-adult sheep and then decreased to adults’ levels. This can be attributed to the high physiological demand for nitrogen for rapid growth during this stage, potentially leading to increased intake of the available forage and thus a greater flow of protein to the rumen [24]. Consequently, proteolysis and deamination rates may be elevated relative to the microbial capacity to incorporate the released ammonia into microbial protein at this specific developmental point. In contrast, the more diverse and complex adult rumen community (Figures 4, 6) might utilize nitrogen more efficiently for microbial synthesis. Furthermore, the increased abundance of carbohydrate-degrading bacteria (e.g., *Succiniclasticum*, *Prevotellaceae_UCG-001*) in adults could enhance the production of bacteriocins or alter the rumen environment, potentially inhibiting some ammonia-producing bacteria and contributing to the lower NH_3_–N concentration observed in adulthood [25].

SCVFAs are the by-products of rumen fermentation and constitute a major energy source for ruminants [26]. Despite the significant shifts observed in microbial composition and NH_3_-N concentrations across developmental stages, the concentrations of most SCVFAs, including acetate, propionate, and butyrate remained relatively stable. A major contributing factor is the presence of a core microbial community that remains consistent across age groups. According to the Venn diagram analysis, 121 genera (46.7% of total identified genera) were shared among all groups, suggesting a stable core microbiota. Key genera such as *Prevotella_1*, *Selenomonas_1*, and *Treponema_2* which play essential roles in the degradation of complex carbohydrates and the production of SCVFAs were dominant and consistently present across all stages. In addition, the forage consisted largely of salt tolerant and alkali-tolerant plants rich in fiber, with NDF and ADF contents of 63.30% and 36.93%, respectively. This uniform fiber-rich diet provides a constant substrate for fibrolytic bacteria, resulting in steady fermentation activity and consistent SCVFAs output across all age groups.

### The rumen microbiota diversity was more diverse and stable with age

In the current research, rumen microbial diversity, as indicated by the Chao1 index and observed species, was higher in adult SF-sheep compared to young and sub-adult individuals. This finding aligns with previous studies [9], which showed that the rumen microbial communities tend to diversify and gradually stabilize as ruminants reach adulthood. Additionally, rumen microbial network co-occurrence analysis revealed that network topology parameters, such as total nodes, links, avgCC and modularity increased with age. This suggests that the interconnection between microorganisms and each module became more complex, and the stability of the bacterial co-occurrence improved under external environment disturbances [27]. Furthermore, PCoA analysis with a homogeneity of dispersions test found that the bacterial composition of young sheep was significantly different from that of sub-adult and adult sheep, indicating that the structure of rumen microbiota becomes more similar and stable with age. Similar results have been demonstrated by previous studies [28]. The results of LEfSe analysis revealed no significant microbial biomarkers in the young SF-sheep group, further suggesting that the rumen microbiota of adult and sub-adult SF-sheep has matured and formed a relatively stable microbiome structure. In contrast, the intestinal microbial community of lambs may still be in development and flux. Although some genera (e.g., *Brevundimonas*, *Lachnospiraceae_NK4B4_group*, *Oscillibacter*, and *Mannheimia*) were unique to the young group**,** their average relative abundances were exceedingly low (0.002%–0.007%), making it difficult to detect statistically significant biomarker taxa. Furthermore, the functional roles of these genera in early rumen development may be more generalist and redundant, lacking the taxonomic specificity or dominance that typically characterizes biomarker taxa in more developed microbiota.

### Rumen fermentation parameters were associated with rumen microbiota

In this study, the significant correlation between the matrix of fermentation parameters and the genus abundance matrix aligns with findings in Tibetan sheep [10], yak [29], indicating a robust association between the rumen microbial community and fermentation parameters. VPA analysis further identified the key fermentation parameter influencing microbial community structure and showed that age and NH_3_-N exerted the greatest effects on rumen bacteria, suggesting that age is the most critical factor associated with bacterial communities. SCVFAs, the end products of forage fermentation through forage–microbe interactions, include acetate and propionate as the main contributors to ruminant energy and nutrition [30]. RDA analysis revealed a potential relationship between the rumen microbiota and SCVFAs. Notably, the production of acetate and propionate was positively associated to *Prevotella_1*, *Prevotellaceae_UCG_00*1, and *Selenomonas_1*. These findings further demonstrate the involvement of microbes in the production of acetate and propionate [31]. Given the symbiotic associations between specific rumen microbes and SCVFAs, our results have important implications for regulating animal nutrition and health through microbial interactions. However, due to the complex dynamics among microbes, such as resource competition and cross-feeding, it remains challenging to identify which taxa are responsible for particular SCVFAs. Therefore, future studies should focus on isolating bacterial strains and elucidating the associations between specific microbes and metabolites.

### The rumen microbial community reflects the different developmental stages and functions of semi-fine wool sheep

Previous studies have shown that high gut microbial diversity is positively related to cellulolytic activity [32] and improves forage apparently digestibility [11]. In our study, compared with young SF-sheep, the higher alpha diversity observed in sub-adult and adult SF-sheep may improve the degradation of plant cellulose and polysaccharides. Our results also indicated that Firmicutes and Bacteroidetes are the two predominant phyla, with Firmicutes were abundant in young SF-sheep, while Bacteroidetes were more enriched in adult sheep. This founding is partly in consistent with Jami et al [9] and Liu et al [14] who reported an age-related increase in the relative abundance of Firmicutes and Bacteroidetes in ruminants. At the genus level, *Prevotella_1*, *Selenomonas*, and several unidentified genera from the *Prevotellaceae* family were enriched in adult SF-sheep. Some of these genera, such as *Prevotella_1*, *Prevotellaceae_UCG-003*, *Prevotellaceae_UCG-003*, were prevalent not only in SF-sheep but also in Tibetan sheep [10]. These bacterial groups are involved in degrading cellulose, hemicellulose, and chitin [33], potentially facilitating grazing ruminants’ adaptation to the high-fiber plant diet on the QTP. Additionally, the distinct microbial composition of the rumen at different ages, particularly in lambs, warrants further attention. The genera *Mannheimia* and *Lachnoclostridium* were enriched in lambs. Some *Mannheimia* (eg., *Mannheimia haemolytica*) are pathogens that cause acute septicemia in newborn lambs [34]. Therefore, we speculate that the period from birth to yearling represents a high-risk stage for acute septicemia in SF-sheep, and herdsmen should take preventive measures against this disease.

Our study also identified two distinct enterotypes in SF-sheep, characterized by unique microbial compositions and functional profiles. Enterotype 1 was more prevalent in young SF-sheep, whereas Enterotype 2 was associated with older individuals. This age-dependent distribution suggests that enterotypes are not static but evolve in response to the host’s developmental needs and dietary adaptations. The dominance of *Prevotella_1* in Enterotype 1 aligns with its established role in carbohydrate metabolism and SCVFA production, which are critical for energy acquisition in growing lambs. By contrast, the enrichment of *Ruminococcaceae_UCG_14* in enterotype 2 reflects a shift toward fiber degradation and more complex metabolic pathways in adult sheep, consistent with their reliance on high-fiber, salt-tolerant plants in the QTP environment. Furthermore, functional of microbial pathways revealed significant differences between enterotypes. Enterotype 1 exhibited a higher abundance of KOs involved in acetate, propionate, and butyrate synthesis (e.g., K00656, K00239, K01966), highlighting its role in supporting rapid energy production in young, fast-growing sheep.

## CONCLUSION

The microbial community structure in SF-sheep showed clear age-related variation, while rumen fermentation parameters remained stable across developmental stages. Age strongly shapes the rumen microbiota: with maturation, microbial diversity and interactions increase, producing a more stable community in adulthood. Across growth stages, the host adapts to its feeding environment by restructuring the composition, dominant bacterial taxa, and potential functions of the rumen microbiota. These findings enhance our understanding of the role of rumen microbiota in Qinghai SF-sheep and provide valuable information for microbial-mediated nutritional adaptations nutritional regulation and production development in this breed.

## Figures and Tables

**Table 1:** Topological properties of bacterial co-occurrence networs across different developmental stages

Index	Node	Link	Avg CC	Avg D	Avg PL	Modularity
LS	278	397	0.325	1.43	7.55	0.94
SS	336	826	0.56	2.46	4.79	0.69
AS	336	683	0.61	2.03	2.3	1.32

The ages of 2-month-old, yearling, and 50-month-old semi-fine wool sheep (SF-sheep) are abbreviated as LS, AS, and SS, respectively.

Avg CC, average clustering coefficient; Avg D, average degree; Avg PL, average path length.

## Data Availability

The raw sequencing data reported in this article have been deposited in the Genome Sequence Archive at the China National Center for Bioinformation’s National Genomics Data Center, with the accession number CRA007942.

## SUPPLEMENTARY MATERIAL

**Supplement 1.** The significance analysis of the top four representative bacterial taxa, whose cumulative contribution exceeds 70%, is presented.
