# Supplementary material for: Rumen microbiota and fermentation parameters in Tibetan semi-fine wool sheep reflect growth stages and potential nutritional adaptations
Source: Anim Biosci. 2026 Feb 6;39(6):250616. doi: 10.5713/ab.250616 (PMC13243931; doi:10.5713/ab.250616)
Supplement: Supplementary file 1 [file ab-250616-Supplementary-1.pdf]

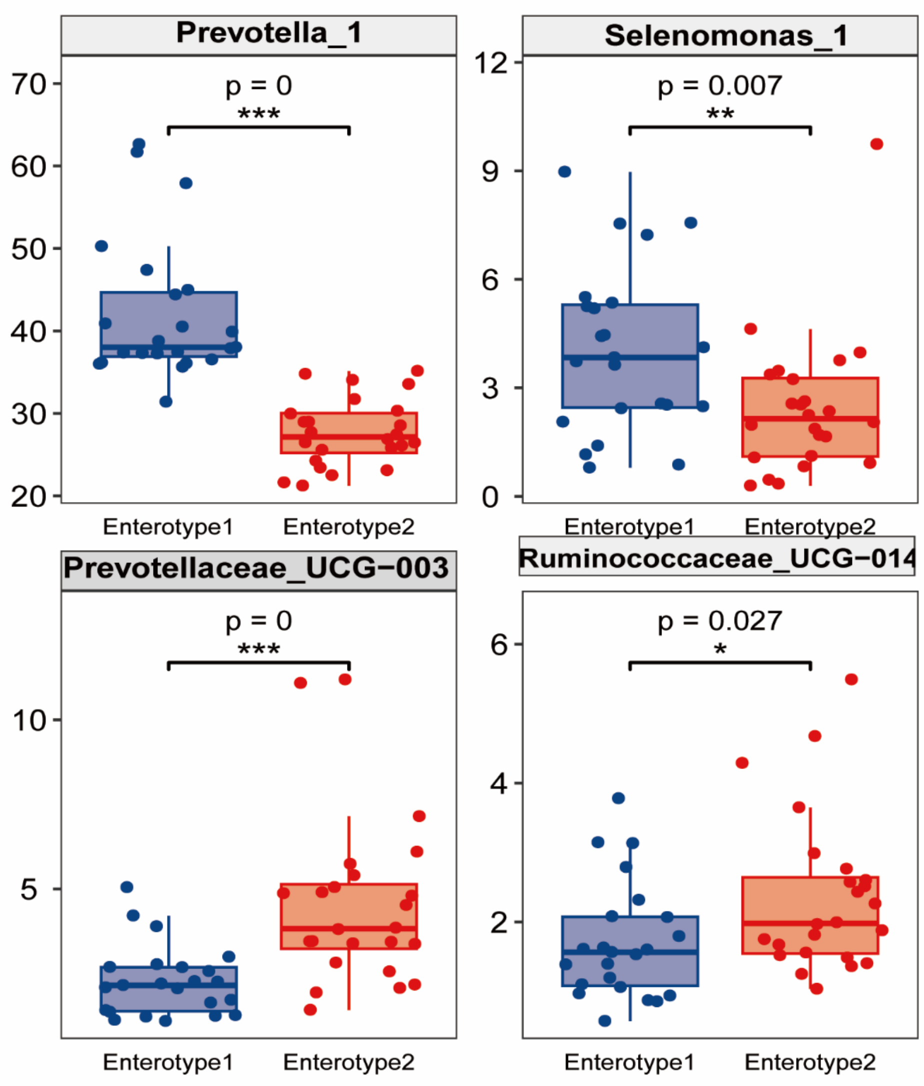

Supplement 1. The significance analysis of the top four representative bacterial taxa, whose cumulative contribution exceeds 70%, is presented. All bar distributions were tested using the Mann–Whitney U test with FDR-corrected p-values. Center values indicate the median, and error bars represent variability.*$p < 0.05$, **$p < 0.01$, ***$p < 0.001$.
